# Staurosporine-induced collapse of cochlear hair bundles

**DOI:** 10.1002/cne.23597

**Published:** 2014-04-17

**Authors:** Richard J Goodyear, Helen SK Ratnayaka, Mark E Warchol, Guy P Richardson

**Affiliations:** 1 Sussex Neuroscience and School of Life Sciences, University of SussexBrighton, BN1 9QG, UK; 2 Department of Otolaryngology, Washington University School of MedicineSt. Louis, Missouri, 63110, USA

**Keywords:** stereocilia, protein kinase inhibitor, radixin, AB_261933, AB_823497, AB_430875

## Abstract

Early postnatal mouse cochlear cultures were treated with a small panel of kinase inhibitors to elucidate the mechanisms underlying the maintenance of hair-bundle structure in the developing inner ear. At low concentrations (1–10 nM), staurosporine causes the collapse and loss of hair bundles without provoking hair-cell death, as judged by lack of terminal transferase dUTP nick end labeling (TUNEL) labeling or reactivity to anti-activated caspase-3. Staurosporine exposure results in the fusion of the hair bundle’s stereocilia, a resorption of the parallel actin bundles of the stereocilia into the cytoplasm of the hair cell, a detachment of the apical, non-stereociliary membrane of the hair cell from the underlying cuticular plate, and a severing of the hair-bundle’s rootlets from the actin cores of the stereocilia. It does not block membrane retrieval at the apical pole of the hair cells, nor does it elicit the externalization of phosphatidylserine. Staurosporine treatment causes a reduction in levels of the phosphorylated forms of ezrin, radixin, and moesin in cochlear cultures during the period of hair-bundle loss, indicating the integrity of the hair bundle may be actively maintained by the phosphorylation status of these proteins. J. Comp. Neurol. 522:3281–3294, 2014. © 2014 Wiley Periodicals, Inc.

Hair cells are found in the vertebrate inner ear and in the lateral line organs of fishes and amphibia. A distinguishing and essential feature of the hair cell is the hair bundle, a mechanosensory structure that is located at the cell’s apical pole. The hair bundle is composed of two or more rows of height-ranked stereocilia and, in most organs, a single eccentrically placed kinocilium that is located next to the tallest stereocilia. Deflections of the hair bundle along one axis, towards or away from the tallest row of stereocilia, are transduced into electrical signals that are then propagated, via the afferent dendrites of the spiral neurons that contact the basal pole of each hair cell, to the central nervous system.

The stereocilia of the hair bundle are rigid, pencil-like processes that are packed with a paracrystalline array of uniformly polarized and highly cross-linked actin filaments (Flock and Cheung, 1977; DeRosier et al., 1980). The stereocilia taper around their basal ends, with a proportion of the actin filaments forming rootlets that project through the tapered region down into the cuticular plate, a dense cytoskeletal meshwork that lies just below the hair bundle in the apical cytoplasm of the hair cell. These elements of the hair bundle are interconnected via a variety of cell-surface specializations known as links or connectors. Up to four distinguishable link types (tip links, horizontal top connectors, shaft connectors, and ankle links) can bridge the membranes of adjacent stereocilia, and kinociliary links couple the kinocilium, when present, to its neighboring stereocilia (Nayak et al., 2007).

The senses of hearing and balance depend critically on the normal development and continual maintenance of the hair bundle. Mutations in a large number of genes cause deafness and balance disorders and, in many instances, it is the structure and/or function of the hair bundle that is affected (Frolenkov et al., 2004; Petit and Richardson, 2009; Schwander et al., 2010). The proteins encoded by these genes include cell-surface molecules, myosin motors, scaffolding proteins, actin cross-linkers, actin-membrane linkers, and proteins required for intraflagellar transport and the establishment of planar polarity. Quite how the activity of all these proteins is orchestrated to produce and maintain a precisely organized hair bundle remains, however, unclear. As many cellular processes are controlled by the phosphorylation status of proteins and lipids, we screened a number of characterized kinase inhibitors for their ability to perturb the structure of hair bundles in organotypic cultures of the early postnatal mouse cochlea.

One compound, staurosporine, was found to cause the collapse and resorption of auditory hair bundles throughout the length of the cochlea without provoking hair-cell death. Staurosporine also reduces the levels of the phospho-ERM proteins (ezrin, radixin, and moesin) in cochlear cultures and its effects phenocopy those that have been observed in the hair cells of radixin knockout mice at later stages of development (Kitajiri et al., 2004).

## MATERIALS AND METHODS

### Reagents

All inhibitors were obtained from Calbiochem (Nottingham, UK). Rhodamine phalloidin, polyclonal antibody to radixin, Dulbecco’s modified Eagle’s medium (DMEM)/F12, 1 M Hepes buffer, and ampicillin were from Sigma (Gillingham, UK). Antibodies to phospho-ERM proteins were from New England Biolabs (Hitchin, UK), Alexa-Fluor 488 goat antirabbit IgG and phalloidin conjugates were from Life Technologies (Paisley, UK). Details of primary antibody immunogens and their source are presented in Table1. The terminal transferase dUTP nick end labeling (TUNEL) labeling kit was from Merck-Millipore (Watford, UK). Collagen was from Becton Dickinson (Oxford, UK) and fetal bovine serum was from Biosera (Uckfield, UK) or PAA (Yeovil, UK). Hank’s balanced salt solution was from Life Technologies (Paisley, UK). Reagents for electron microscopy were obtained from TAAB (Berks, UK).

**Table 1:** Summary of Antibodies Used

Name	Immunogen	Commercial supplier	Details	RRID
Radixin	Residues 400–409, Lys-Ser-Ala-Ile-Ala-Lys-Gln-Ala-Ala-Asp, of human radixin	Sigma-Aldrich, R3653	Rabbit polyclonal	AB_261933
Phospho-ERM	Synthetic peptide Gly-Arg-Asp-Lys-Tyr-Lys-phosphoThr-Leu-Arg-Gln-Ile-Arg corresponding to amino acids 561–572 of human ezrin	Cell Signaling, 3149S	Rabbit monoclonal	AB_823497
Activated caspase-3	Peptide derived from the 29–175 chain of the caspase-3 subunit (p17 fragment of human caspase-3) with sequence homology in mouse, rat and hamster.	Promega, G7481	Rabbit polyclonal	AB_430875

### Preparation of mouse cochlear cultures

Cochlear cultures were prepared from CD1 mice at postnatal day (P) 1 or P2 using methods described previously (Russell and Richardson, 1987). The cochlear coils were dissected in HEPES-buffered (10 mM pH 7.2) Hanks’ balanced salt solution (HBHBSS), plated onto hydrated collagen gels that had been prepared on glass coverslips, fed with 1–2 drops (50–100 μl) of medium (93% DMEM/F12 HAM, 7% fetal bovine serum, 10 μg/ml ampicillin), sealed into Maximow slide assemblies, and grown for 24 hours at 37°C.

### Treatment of cochlear cultures with kinase inhibitors

Coverslips were removed from the Maximow slide assemblies, placed in 35-mm diameter plastic Petri dishes, washed once with HBHBSS, and incubated with control medium or medium containing the inhibitor to be tested. Inhibitors were obtained or prepared as stock concentrates (usually at a concentration 100–1,000× greater than that required) in dimethyl sulfoxide (DMSO) or H_2_O. An equivalent volume of DMSO or H_2_O was added to the control medium. After 5–48 hours incubation at 37°C, cultures were fixed in 4% paraformaldehyde (Agar Scientific, Essex, UK) in 0.1 M sodium phosphate buffer, pH 7.4, for 1 hour at room temperature, and then rinsed three times in phosphate-buffered saline (PBS).

### Phalloidin and antibody staining

To detect changes in hair-bundle structure, cultures were stained with 0.5 μg/ml of rhodamine conjugated phalloidin in PBS containing 10% horse serum and 0.1% Triton X-100 (PBS/HS/T) for 1.5 hours at room temperature, rinsed three times in PBS, and mounted on glass slides with Vectashield mounting medium (Vector Laboratories, Peterborough, UK). Slides were viewed with a Zeiss LSM 510 confocal microscope. To label radixin or phosphorylated forms of the ezrin-radixin-moesin family of proteins, cultures were stained overnight at 4°C in rabbit anti-radixin (1:100 dilution) or rabbit anti-phospho-ERM (1:50 dilution), washed three times with PBS, labeled with a mixture of Texas Red phalloidin (Invitrogen, 1:500) and Alexa-488 conjugated goat antirabbit Ig (Invitrogen, 1:500) in PBS/HS/T for 2–3 hours at room temperature, washed, and mounted in Vectashield.

### Antibody Characterization

Western blotting confirmed the specificity of the antibodies used, with the anti-radixin polyclonal rabbit antibody (Sigma-Aldrich, R3653) staining a single band of 75 kDa and the anti-phospho-ERM monoclonal rabbit antibody (Cell Signaling, 3149S) staining three bands of 80, 75, and 70 kDa corresponding, respectively, to ezrin, radixin, and moesin in lysates of control mouse cochleae. The anti-radixin antibody has been validated previously by Valderrama et al. (2012), who showed a loss of labeling in western blots of PC3 cells in which radixin, but not ezrin or moesin, had been specifically depleted by siRNA. The anti-activated caspase-3 antibody (Promega, Southampton, UK; G7481), used as a marker for apoptotic cell death, was shown previously to specifically label HepG2 cells in which apoptosis had been induced by agonistic anti-FAS antibody (Kamada et al., 2005). Gargini et al. (2007) found also that this antibody only labeled apoptotic cells, staining a subset of photoreceptors that were TUNEL-positive in a mouse model of retinal degeneration. Furthermore, in the present study, the anti-activated caspase-3 antibody did not stain control, nondrug-treated cultures, but specifically recognized hair cells judged to be apoptotic on the basis of both TUNEL and DAPI labeling. Further details on immunogens, species in which the antibodies were raised, and RRID numbers are presented in Table1.

### AnnexinV labeling

To study the externalization of phosphatidylserine, cultures were placed in a glass-bottomed Perspex chamber in 0.5 ml HBHBSS and 5 μl of Alexa-Fluor 488-conjugated annexin V (Invitrogen) was added and mixed rapidly. Images were acquired with wide-field microscopy at fixed timepoints (usually at 5–8 and 10–13 minutes) after the addition of annexin V using a Zeiss Axioplan II microscope equipped with a 63× water immersion lens, an FITC filter set, and a 100 W AttoArc HBO mercury vapor lamp. Differential interference contrast images were captured just prior to the fluorescent images.

### TUNEL staining and anti-activated caspase-3 labeling

For activated caspase-3 labeling, cultures were incubated for 8 or 18 hours in medium alone, or medium containing 10 nM staurosporine or 0.5 mM neomycin. Cultures were washed in HBHBSS, fixed, and preblocked as described above and labeled with rabbit anti-activated caspase-3 diluted 1:250 in PBS/HS/T overnight at 4°C followed by secondary antibodies and phalloidin as described earlier. Cultures were mounted in Vectashield containing DAPI. For TUNEL labeling, the protocol of the Apotag Plus fluoroscein in situ apoptosis detection kit was followed with the following modifications. Cultures were initially permeabilized with PBS containing 0.1% Triton X-100 for 20 minutes and cultures were labeled with Alexa-Fluor 647 phalloidin diluted to 1:100 in PBS/HS following the TUNEL procedure.

### Scanning electron microscopy (SEM)

Cultures were fixed in 2.5% glutaraldehyde in 0.1 M sodium cacodylate buffer, pH 7.2, for 2 hours at room temperature, rinsed three times in sodium cacodylate buffer, and postfixed with cacodylate-buffered 1% osmium tetroxide for 1 hour. Tissue pieces were then washed twice in 0.1 M cacodylate buffer, once with H_2_O, and dehydrated through a series of ascending concentrations of ethanol, with the final 100% dehydration step carried out overnight at 4°C. Following critical point drying, samples were mounted on aluminum specimen stubs, sputter-coated with gold particles, and viewed with a Leica Leo S240 scanning electron microscope.

### Light and electron microscopy

Cultures were fixed in 2.5% glutaraldehyde in 0.1 M sodium cacodylate buffer, pH 7.2, containing 1% tannic acid for 2 hours at room temperature, rinsed three times in sodium cacodylate buffer, and postfixed with cacodylate-buffered 1% osmium tetroxide for 1 hour. Tissue pieces then washed twice in cacodylate buffer, once with H_2_O, dehydrated through a series of ascending concentrations of ethanol (5–10 minutes per step), equilibrated in propylene oxide, and embedded in Taab 812 epoxy resin. Tissue blocks were cured at 60°C for 24 hours. One-micron-thick sections were cut using glass knives, placed on glass slides, stained with Toluidine blue dye for ∼1 minute at ∼60°C, mounted with Histomount, and viewed with a Zeiss Axioskop light microscope. Ultrathin sections (100 and 150 nm thick) were cut on a Reichert Ultracut E microtome, mounted on copper grids, counterstained with 1% aqueous uranyl acetate followed by lead citrate, and viewed in a Hitachi 7100 transmission electron microscope (TEM).

### Cationic ferritin labeling

Staurosporine-treated cultures were washed once with cold HBHBSS, labeled on ice with 1 mg/ml cationic ferritin diluted in cold, serum-free, HEPES-buffered (10 mM, pH 7.2) medium, washed three times in HBHBSS, and then exposed to staurosporine for a further 3 hours at 37°C in medium as described above.

### Western blotting

Cultures were treated with 0.1% DMSO or staurosporine (10 nM) for 1, 2.5, 5, 10, and 14 hours at 37°C, removed from the collagen-coated coverslip, and frozen. Frozen samples were subsequently thawed in reducing sodium dodecyl sulfate-polyacrylamide gel electrophoresis (SDS-PAGE) sample buffer, and heated at 100°C for 5 minutes. Aliquots of the supernatants were run on 10% polyacrylamide gels and the proteins transferred to PVDF membranes using semidry blotting. Protein blots were preblocked in TBS containing 10% horse serum and 0.1% Tween-20 (TBST), reacted overnight with anti-radixin (1:500) or anti-phospho-ERM antibody (1:1,000), washed with TBS, incubated with alkaline phosphatase conjugated goat antirabbit IgG for 2 hours, washed, and reacted with NBT/BCIP for 15 minutes. Blots were washed, dried, scanned, and densitometry was performed using ImageJ software (NIH, Bethesda, MD).

## RESULTS

The compounds tested, their primary targets, the concentrations at which they were used, the durations for which they were applied, and whether or not they affected hair-bundle structure are summarized in Table2. Most inhibitors were tested at concentrations corresponding to the reported IC_50_ values for their primary targets, and at concentrations one or more orders of magnitude higher than the IC_50_. Cochlear cultures were exposed to the compounds for 24–48 hours and, following fixation and staining with rhodamine conjugated phalloidin, analyzed by fluorescence microscopy. Of the protein kinase inhibitors used in this initial screen, the Rho-associated coiled-coil forming protein kinase (ROCK) inhibitor Y-27632, the cGMP-dependent protein kinase (PKG) inhibitor KT5823, Inhibitor IX of glycogen synthase kinase (GSK)-3a/b, and the cJun N-terminal kinase (JNK) inhibitor SP600125 were found to have no effect at the highest concentration at which they were tested. The calmodulin-dependent protein kinase (CaM kinase) inhibitor KN-93, and the myosin light chain kinase inhibitor, ML-7, were toxic to cochlear cultures at high concentrations, and were without effect at 5-10-fold lower concentrations.

**Table 2:** List of Compounds Tested, Their Targets, the Concentrations and Times Used, and Whether or Not They Altered Hair-Bundle Structure, or Showed Toxicity

Compound	Target	Concentration	Time (hrs)	Effect on hair bundles
AKT Inhibitor IV	AKT/PKB	1 µM	24	Toxic
		5 µM	24	Toxic
KN-93	CaM kinase	740 nM	43	No
		5 µM	24	Toxic
		50 µM	24	Toxic
GSK-3 inhibitor IX	GSK-3α/β	25 nM	24, 39	No
		50 nM	24	No
		100 nM	24	No
Staurosporine	Multiple kinases	1 nM	5, 6, 10, 15, 20, 24, 48	Yes
		5 nM	16	Yes
		10 nM	14, 16, 24, 48	Yes
		20 nM	24, 48	Yes
		100 nM	24	Yes
		200 nM	24, 48	Yes
		1000 nM	24, 48	Yes
ML7	MLCK	600 nM	43	No
		1 µM	24, 48	No
		10 µM	24, 48	No
		50 µM	24, 48	Toxic
LY294002	PI 3-kinase	5 µM	24	No
		10 µM	24	Yes
		20 µM	24, 48	Yes
Wortmannin	PI 3-kinase	20 nM	24, 48	Yes
H89	PKA	96 nM	43	No
		10 µM	24	Yes, apical
		100 µM	24	Toxic
Bisindolylmaleimide I (BIM I)	PKC α, β1, β2, δ, ε, γ isoenzymes	20 nM	43	No
		100 nM	24	No
		1000 nM	24	No
Rottlerin	PKC δ, θ isoenzymes	1 µM	24	No
		2 µM	24	No
		5 µM	24	Yes
Myristoylated pseudosubstrate peptide inhibitor	PKC θ	1 µM	24	No
		10 µM	24	No
		40 µM	24	Toxic
KT5823	PKG	468 nM	43	No
		1 µM	24	No
		5 µM	24	No
		10 µM	24	No
C3 transferase	Rho A, B, C	2 µg/ml	6, 14	No
		4 µg/ml	6	No
Y27632	ROCK	500 nM	24, 48	No
		5 µM	24, 48	No
		50 µM	24	No

**Figure 1:**
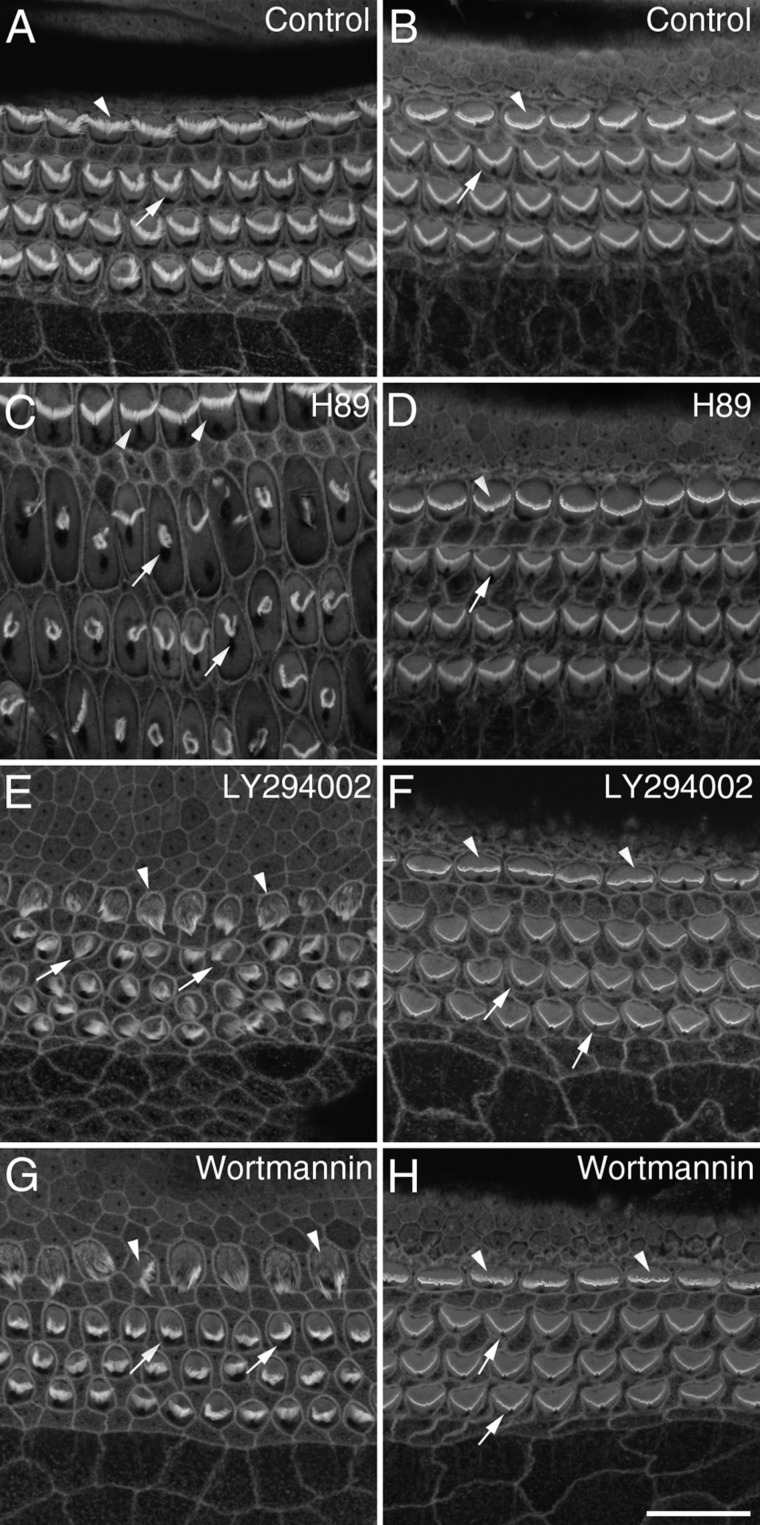
The effects of protein kinase and phosphoinositide 3-kinase inhibitors on cochlear cultures. Confocal projections from the mid-apical (A,C,E,G) and mid-basal (B,D,F,H) regions of phalloidin-stained cochlear cultures that were grown in control medium (A,B) or in medium containing 10 μM H89 (C,D), 10 μM LY294002 (E,F), or 20 nM wortmannin (G,H) for 24 hours. Arrowheads point to inner hair cells, arrows point to outer hair cells. Scale bar = 20 μm in H (applies to all).

**Figure 2:**
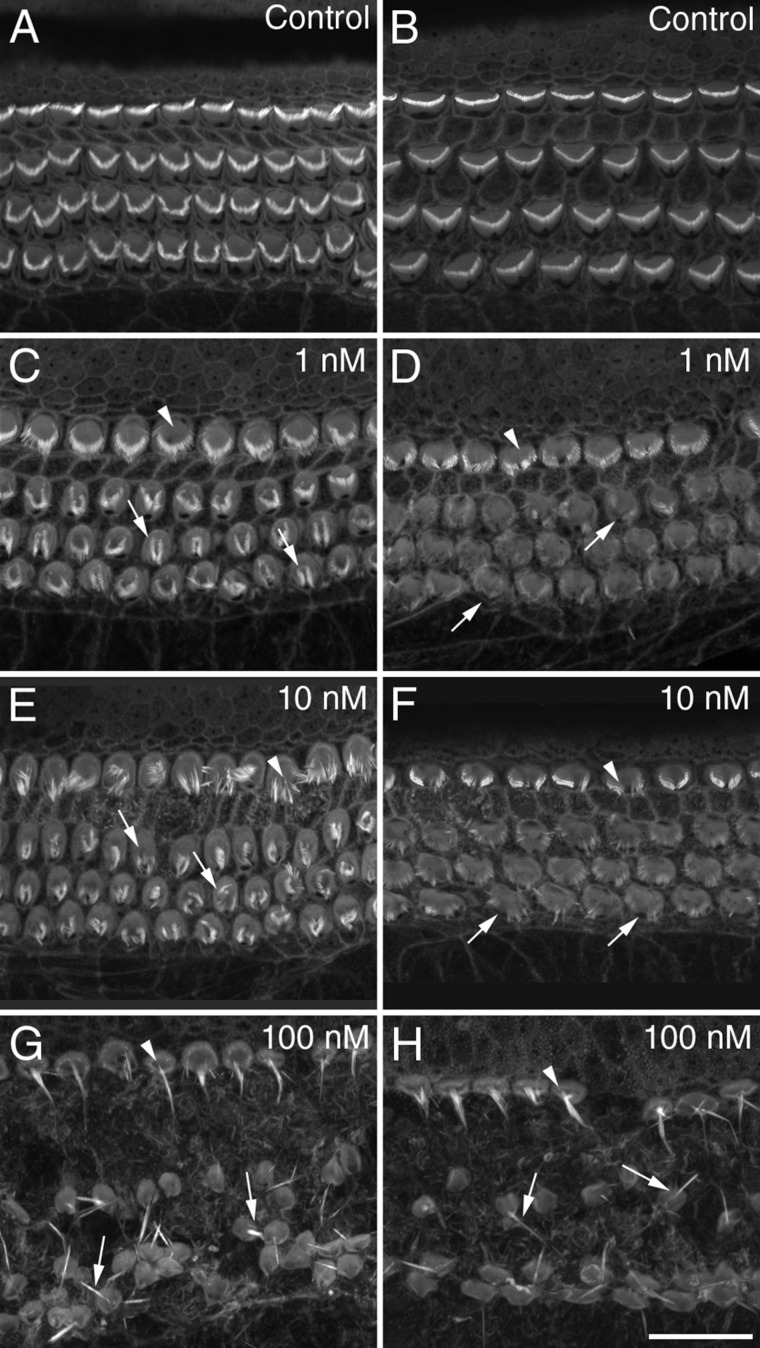
Staurosporine dose–response. Confocal images from mid-apical (A,C,E,G) and basal (B,D,F,H) coil cochlear cultures grown for 16 hours in control medium (A,B) or in medium containing staurosporine at a concentration of 1 nM (C,D), 10 nM (E,F), or 100 nM (G,H). At concentrations of 1 and 10 nM staurosporine, hair bundles appear to partially collapse or splay (E,F), while at 100 nM staurosporine exposure results in severe disruption of the organ of Corti and fusion of stereocilia (G,H). Arrowheads and arrows point to disrupted IHCs and OHCs, respectively, in parts C–H. Scale bar = 20 μm in H (applies to all).

Figure 1 illustrates the morphology of control cultures and the effects of kinase inhibitors that caused disruption of hair-bundle structure without a general toxic effect on the culture. The appearance of phalloidin-stained hair bundles in the middle of apical and basal-coil cochlear cultures following 24 hours in control medium is illustrated in Figure 1A,B. At a concentration of 10 μM, the protein kinase A (PKA) inhibitor H89 caused a considerable expansion of the apical surface of hair cells and a distortion of the typical V-shaped pattern of the outer hair cell (OHC) bundles (Fig. 1C). This effect, however, was restricted to cells in the apical-coil cultures and not observed in basal-coil hair cells (Fig. 1D). Two inhibitors of the lipid kinase phosphatidylinositol 3-kinase (PI-3 kinase), LY294002 and the fungal metabolite wortmannin, also preferentially affected the shape of apical-coil (Fig. 1E,G), as opposed to basal-coil (Fig. 1F,H) cochlear hair bundles. LY294002 resulted in changes to hair-bundle morphology at a concentration of 10 μM (Fig. 1E). At a concentration of 20 nM wortmannin also resulted in some disruption of hair-bundle structure; hair bundles of OHC became more compact in appearance, and those of the inner hair cells (IHC) became severely distorted (Fig. 1G). Only one of the inhibitors tested, staurosporine, caused the collapse of hair bundles throughout the entire length of the cochlea (Fig. 2). Its effects were therefore examined in more detail.

### Staurosporine: effects of time and concentration

Concentrations of staurosporine ranging from 1 to 100 nM were tested for a period of 16 hours on both apical and basal-coil cochlear cultures. Staurosporine caused a distinct change in hair-bundle morphology in both apical and basal-coil cochlear cultures at concentrations as low as 1 nM (Fig. 2A–D). At 10 nM the effects were comparable, although more pronounced (Fig. 2E,F) with collapse of both IHC and OHC bundles being the most noticeable feature of drug treatment. At 100 nM, hair-bundle morphology changed dramatically with fused, elongated hair bundles predominating in all regions (Fig. 2G,H).

**Figure 3:**
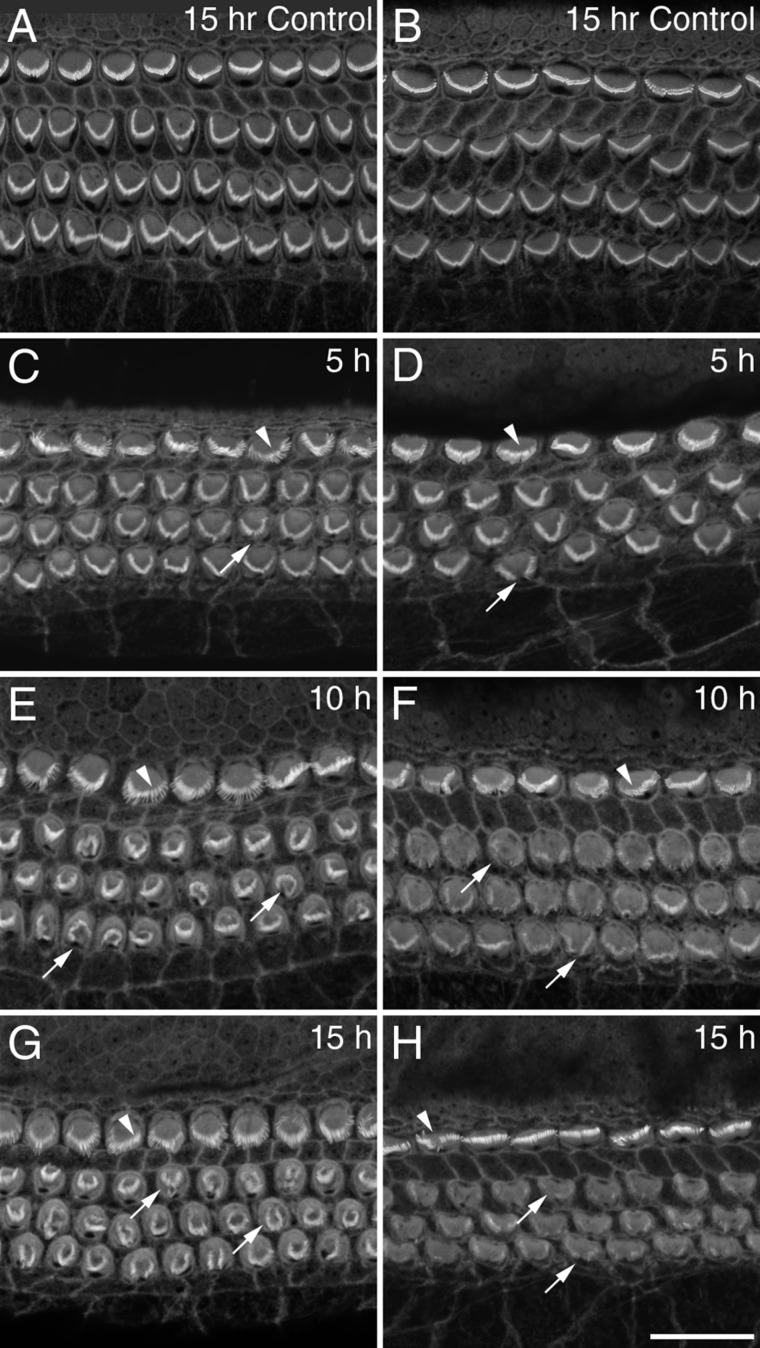
Staurosporine time course. Confocal images from mid-apical (A,C,E,G) and mid-basal (B,D,F,H) regions of cochlear cultures grown for 15 hours in control medium (A,B) or medium containing 1 nM staurosporine for 5 (C,D), 10 (E,F), or 15 (G,H) hours. Although partial collapse of hair bundles is first observed after 5 hours of staurosporine (C,D), the disruption is much more pronounced by 10 hours (E,F). No obvious progression is observed after a further 5 hours (G,H). Arrowheads point to examples of IHCs and arrows point to examples of OHCs that show signs of disruption. Scale bar = 20 μm in H (applies to all).

In order to observe the time course of staurosporine damage, cochlear cultures were treated with 1 nM staurosporine and fixed after 5, 10, and 15 hours of treatment (Fig. 3A–H). Some subtle but noticeable effects of the drug were observable after 5 hours, with a partial collapse of hair bundles occurring (Fig. 3A–D). After 10 hours of staurosporine treatment, hair-bundle collapse was much more apparent (Fig. 3E,F). By 15 hours of treatment the effects of staurosporine were not noticeably worse (Fig. 3G,H).

### Staurosporine: ultrastructural effects

**Figure 4:**
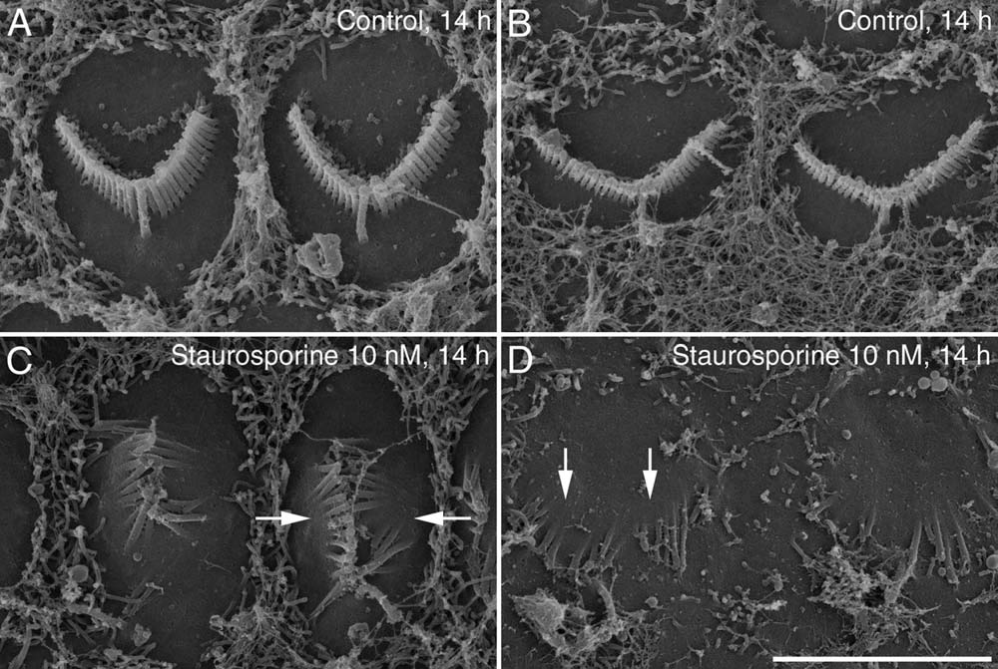
SEM imaging of staurosporine-treated hair bundles. Scanning electron micrographs of hair bundles from mid-apical (A,C) and mid-basal (B,D) OHCs in cochlear cultures that were grown for 14 hours in control medium (A,B) or medium containing 10 nM staurosporine (C,D). Treatment with 10 nM staurosporine for 14 hours typically results in the inward collapse of apical OHC bundles (C, indicated by arrows) and the outward collapse of basal OHC bundles (D, indicated by arrows). Scale bar = 5 μm in D (applies to all).

Scanning electron microscopy of untreated control cultures and cultures that had been exposed to 10 nM staurosporine for 14 hours revealed region-specific variations in the hair-bundle response (Fig. 4A–D). OHC bundles from mid apical-coil cultures frequently collapsed inwards on themselves (Fig. 4C), while those of the mid-basal coil OHCs invariably collapsed outward (Fig. 4D). A conspicuous reduction in the density of microvilli on the apical surfaces of the nonsensory supporting cells surrounding the tops of the hair cells was also noted, especially in the staurosporine-treated basal-coil cultures (Fig. 4D).

**Figure 5:**
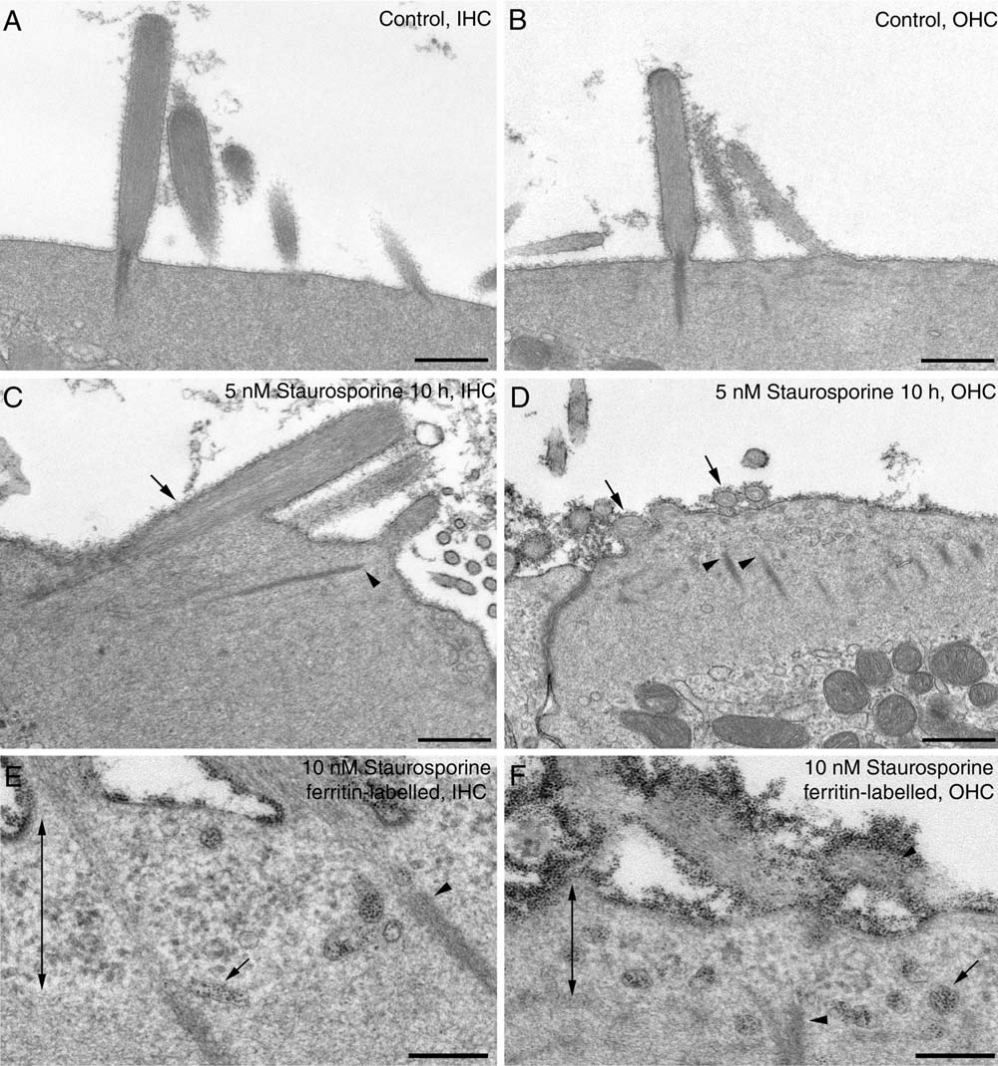
TEM of staurosporine-treated hair cells. TEMs of IHCs (A,C,E) and OHCs (B,D,F) after growth in control medium for 10 hours (A,B) or medium containing 5 nM staurosporine for 10 hours (C,D). In (E,F) cultures were incubated in medium containing 10 nM staurosporine for 4 hours, labeled with cationic ferritin at 4°C for 1 hour, and then incubated for a further 4 hours in medium containing 10 nM staurosporine. Staurosporine causes collapse of stereocilia (arrows in C,D), dissociation of the rootlets from the stereocilia (arrowheads in C–F) and a detachment of the cuticular plate from the apical membrane (double-headed arrows in E,F) with the accumulation of cationic ferritin-labeled vesicles in the region below the stereocilia (small arrows in E,F). Scale bars = 500 nm in A–D; 200 nm in E,F.

Transmission electron microscopy revealed that staurosporine caused the stereociliary rootlets to become thinned or even severed from their associated stereocilia (Fig. 5A–F). Furthermore, the apical plasma membrane was detached from the underlying cuticular plate and numerous vesicles and other cellular components were observed between the hair-cell surface and the severed end of the rootlets (Fig. 5A–F). To determine if the vesicles observed in this region were involved in apical membrane turnover, cultures were labeled with cationic ferritin during treatment with staurosporine. Numerous ferritin-labeled vesicles were observed in the region where the apical membrane had detached from the cuticular plate (Fig. 5E,F). Cationic ferritin-labeled vesicles were also present, as in control cultures, in the region of the pericuticular necklace in staurosporine-treated cultures (not shown). The observed increase in cationic ferritin-labeled vesicles in the region of cuticular plate detachment suggests that this structure may normally restrict membrane retrieval to the more peripheral regions of the hair cell’s apical surface.

### Low concentrations of staurosporine do not kill hair cells

**Figure 6:**
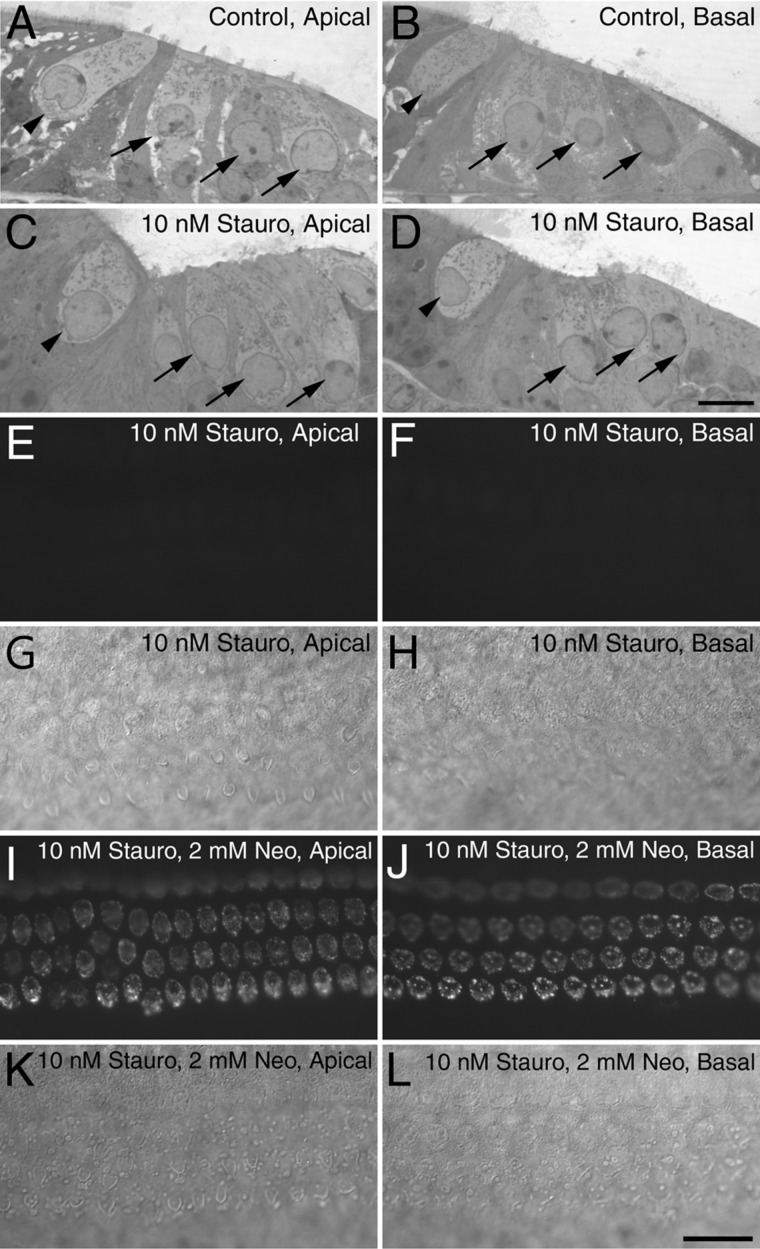
The effects of staurosporine on hair-cell survival. Semithin Toluidine blue-stained sections of cultures grown in medium alone (A,B) or medium containing 10 nM staurosporine (C,D) for 48 hours. Hair cells are still present in staurosporine-treated cultures (C,D). Arrowheads point to IHCs, arrows point to OHCs in A–D. PS externalization is not seen following Alexa-488 annexin-V labeling of cochlear cultures that were grown in medium containing 10 nM staurosporine (E,F) for 8 hours. Staurosporine treatment does not prevent the subsequent brief application of neomycin from eliciting PS externalization (I–J). Corresponding DIC images for (E,F,I,J) are shown in (G,H,K,L). Scale bars = 10 μm in D (applies to A–D); 20 μm in L (applies to E–L).

**Figure 7:**
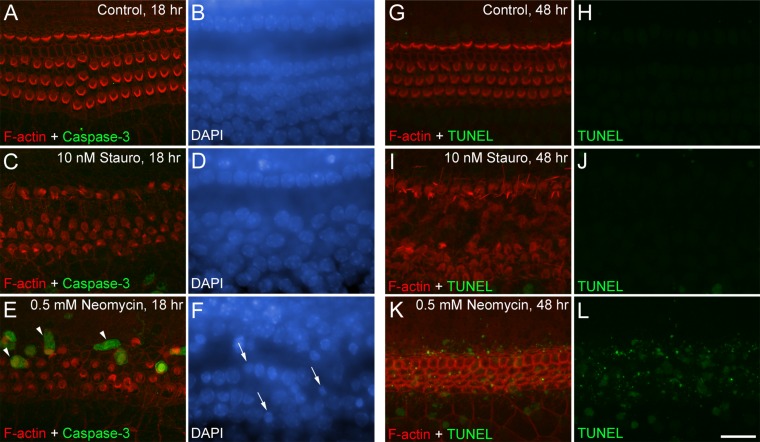
Activated caspase-3 labeling and TUNEL in staurosporine-treated cultures. Phalloidin (red) and anti-activated caspase-3 (green) staining following treatment with control medium (A) or medium containing 10 nM staurosporine (C) or 0.5 mM neomycin (E) for 18 hours. Images A–C are confocal Z-compressions encompassing a depth of 15 μm below the apical surface and are taken from the mid-apical region. Caspase-3 positive hair cells (arrowheads in E) are only observed in the neomycin condition. Corresponding DAPI-stained nuclei are shown in (B,D,F). Although hair-cell nuclei of staurosporine-treated cultures have a normal appearance, those exposed to neomycin are often shrunken and condensed (arrows in F). Phalloidin staining (red) and TUNEL (green) following treatment with control medium (G,H) or medium containing 10 nM staurosporine (I,J) or 0.5 mM neomycin (K,L) for 48 hours. Images are confocal Z-compressions encompassing a depth of 15 μm below the apical surface and are taken from the mid-apical region. TUNEL-positive hair-cell nuclei are abundant following neomycin but not staurosporine. A magenta-green version of this figure is available as Supporting Figure 1. Scale bar = 20 μm.

Cochlear cultures were treated for 48 hours with 10 nM staurosporine and then fixed and sectioned in order to see if the drug caused the loss of hair cells. Inner and outer hair cells and their hair bundles were readily visible in Toluidine-blue stained 1-μm thick sections from the apical and basal coils of control cultures (Fig. 6A,B). Although hair bundles were not seen in the staurosporine-exposed cultures, hair-cell bodies remained within the epithelium and had nuclei of normal appearance (Fig. 6C,D) in both the apical and the basal coils. One of the most rapid known effects of ototoxins like the aminoglycoside antibiotics is to cause the externalization of phosphatidylserine at the hair cell’s apical pole (Goodyear et al., 2008). Cultures were therefore treated with 10 nM staurosporine for 8 hours at 37°C and then exposed to Alexa-Fluor 488-conjugated annexin V to determine if phosphatidylserine was present on the external surface of the hair cells. Control cultures did not label with annexin V (not shown), nor did cultures that were exposed to 10 nM staurosporine (Fig. 6E,F). By contrast, exposure to 2 mM neomycin, a known ototoxin that selectively kills hair cell in cochlear cultures, elicited PS externalization in both the apical and basal coils of staurosporine-treated cultures within 10 minutes (Fig. 6I,J). To confirm hair cells were not undergoing apoptosis in response to staurosporine treatment, cultures were treated with 10 nM staurosporine or 0.5 mM neomycin for 18 or 48 hours, fixed, and either double-labeled with the nuclear dye DAPI and antibodies to activated caspase-3, or subjected to TUNEL (Fig. 7). In the cultures treated with neomycin, a proportion of the hair cells were stained by anti-activated caspase-3 (Fig. 7E) and the hair-cell nuclei were of a condensed morphology (Fig. 7F) and positive for TUNEL (Fig. 7K,L). In the staurosporine-treated cultures, activated caspase-3 staining was not observed in hair cells (Fig. 7C), and the hair-cell nuclei were of normal morphology (i.e., not fragmented or condensed) (Fig. 7D) and were not TUNEL-positive (Fig. 7I,J).

### Staurosporine reduces apical cell surface phospho-ERM levels

**Figure 8:**
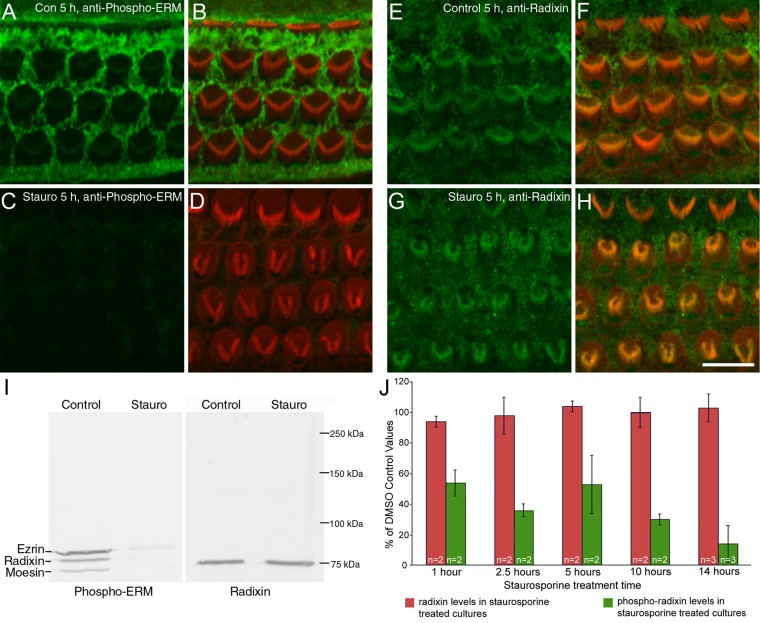
Phospho-ERM levels in staurosporine-treated cochlear cultures. Immunolabeling with anti-phospho-ERM (A–D) and anti-radixin (E–H) antibodies following incubation in control medium (A,B,E,F) or medium containing 10 nM staurosporine (C,D,G,H) for 5 hours. Phalloidin staining is also shown in (B,D,F,H). Staurosporine causes a dramatic loss of phospho-ERM (C) but not of radixin labeling (G). I: Western blots of lysates from cochlear cultures stained for phospho-ERM or radixin following 14 hours in control medium or medium containing 10 nM staurosporine. J: Quantitative analysis of levels of radixin (red bars) and phospho-radixin (green bars) in cochlear cultures that were treated with 10 nM staurosporine for 1, 2.5, 5, 10, and 14 hours. Numbers of independent experiments are indicated. A magenta-green version of this figure is available as Supporting Figure 2. Scale bar = 10 μm in H (applies to A–H).

The effects of staurosporine observed by transmission electron microscopy had, in some instances, a striking resemblance to the changes in hair-bundle structure described in radixin null mutant mice (see fig. 5 in Kitajiri et al., 2004). The distribution of radixin and phosphorylated forms of the ERM proteins were therefore examined in control cultures and cultures that had been treated with 10 nM staurosporine for 5 hours (Fig. 8). Strong labeling of the microvilli on the surfaces of the supporting cells and a much weaker labeling of hair bundles was observed in control cultures with antibodies to phospho-ERM (Fig. 8A). Staurosporine treatment resulted in almost complete loss of all labeling (Fig. 8C). A polyclonal antibody to human radixin stained the sensory hair bundles and the microvilli of the surrounding supporting cells (Fig. 8E), and this distribution remained unchanged in staurosporine-treated cultures (Fig. 8G) despite collapse of the hair bundles.

The presence of phosphorylated ERM proteins and radixin in cochlear cultures that had been exposed to 10 nM staurosporine was examined by western blotting (Fig. 8I). Labeling of phospho-ERM reactive bands was considerably reduced in staurosporine-treated cultures relative to that observed in untreated control cultures. The levels of radixin (phosphorylated + nonphosphorylated), as detected with a polyclonal anti-radixin antibody, were unaffected by treatment with staurosporine (Fig. 8I). Quantitative analysis of western blots indicates that phospho-radixin levels in cochlear cultures are reduced by nearly 50% within 1 hour of staurosporine exposure and decline to less than 20% within 14 hours (Fig. 8J).

**Figure 9:**
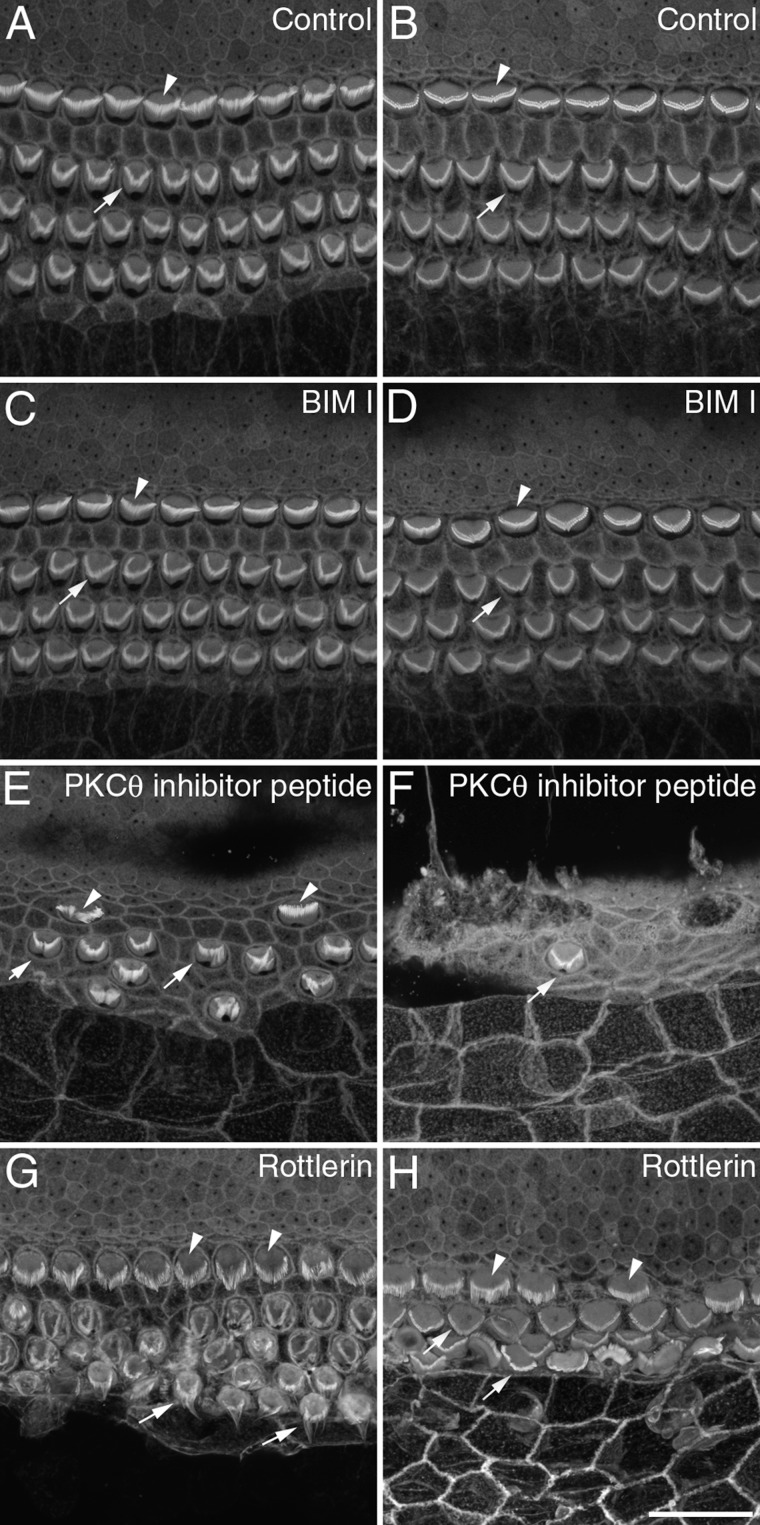
Effects of PKC inhibitors on hair-bundle structure. Confocal images from mid-apical (A,C,E,G) and mid-basal (B,D,F,H) regions of cochlear cultures that were grown for 24 hours in control medium (A,B) or medium containing either BIM I (C,D), PKCθ pseudosubstrate inhibitor (E,F) or rottlerin (G,H). Arrowheads indicate inner hair cells bundles, arrows point to outer hair cell bundles. Scale bar = 20 μm in H (applies to all).

Although staurosporine is a broad-spectrum kinase inhibitor, it has a high affinity for PKC and the PKC isoforms α and θ are candidate ERM kinases (Pietromonaco et al., 1998; Ng et al., 2001). Cochlear cultures were therefore treated with bisindolylmaleimide I (BIM I), an inhibitor of the α, β1, β2, γ, δ, and ε isoenzymes, a myristoylated pseudosubstrate peptide inhibitor of PKCθ, and rottlerin, an inhibitor of the PKC δ and θ isoenzymes (Fig. 9). BIM I was without an effect even at a concentration of 1 μM (Fig. 9C,D). The PKCθ pseudosubstrate inhibitor was without effect at 10 μM, but caused extensive loss of hair cells at 40 μM. While there was an obvious loss of hair cells at 40 μM, those remaining had fairly normal-looking hair bundles that did not resemble those seen following staurosporine treatment (Fig. 9E,F). Rottlerin, at a concentration of 5 μM, caused the loss of some hair cells in both the apical and basal-coil cultures, and caused a considerable distortion of hair-bundle structure, but this was mainly restricted to the apical-coil hair cells (Fig. 9G,H). At a 10-fold higher concentration rottlerin was toxic.

Hair cells in mouse cochlear cultures (Sobkowicz et al., 1997) and those in the cultures of the bullfrog saccule (Gale et al., 2002) have been reported to be able to repair or replace their hair bundles following sublethal insults. In order to determine if mouse cochlear hair bundles could be repaired or regenerated following staurosporine-induced collapse, cultures were exposed to 10 nM staurosporine for 5 hours at 37°C, washed three times with medium, and incubated in normal, staurosporine-free medium at 37°C for a further 18 hours or 4 days, prior to fixation and staining with Texas Red phalloidin. Extensive hair-bundle collapse was observed throughout the cultures that were fixed 18 hours after a 5-hour exposure to 10 nM staurosporine (Fig. 10B). While the damaged hair cells were able to survive for 4 days (Fig. 10D), there was no indication that the initial effects of staurosporine could be reversed, nor was there any evidence for the appearance of immature, newly-formed hair bundles on the apical surface of the drug-treated hair cells (Fig. 10D).

**Figure:**
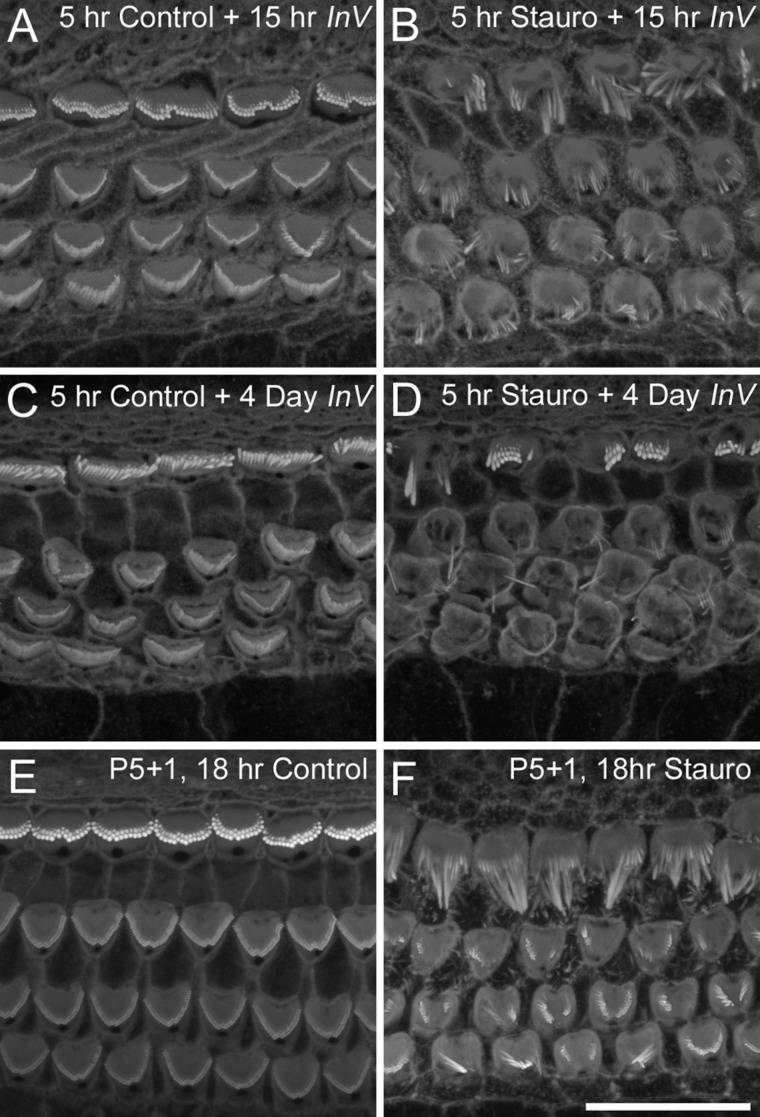
The effects of staurosporine on mature hair cells. Phalloidin staining following 5 hour treatment of P2+1 day in vitro cultures with control medium (A,C) or medium containing 10 nM staurosporine (B,D) that were maintained in vitro for a further 15 hours (A,B) or 4 days (C,D). Images A–D are from the mid-basal region. Phalloidin-staining following treatment of P5+1 day in vitro cultures for 18 hours with control medium (E) or with medium containing 10 nM staurosporine (F). Images E,F are from the middle region of the cochlea. Scale bar = 20 μm.

Hair cells in cochlear cultures that have been prepared from P2 mice are in the process of maturation. In order to determine if staurosporine causes the collapse of more mature hair bundles, cochlear cultures were prepared from P5 mice and, after 1 day in vitro (equivalent to P6), exposed to 10 nM staurosporine for 18 hours. As with cultures prepared from earlier stages of development, hair-bundle collapse was observed in the staurosporine-treated cultures (Fig. 10F), but not in controls exposed to the DMSO vehicle (Fig. 10E).

## DISCUSSION

The results of this study reveal that staurosporine causes a collapse of the stereociliary bundles of sensory hair cells in cochlear cultures prepared from early postnatal mice. Both threshold (1 nM) and supra-threshold (10 nM) concentrations of staurosporine were found to have a selective effect on the stereociliary bundle of the hair cell, and did not cause hair-cell death as judged by a lack of PS externalization, activated caspase-3 staining, and TUNEL. Kinase inhibitors that had a general toxic effect on organotypic cochlear cultures and could not be evaluated included the CaM kinase inhibitor KN-93, the myosin light chain kinase inhibitor ML-7, and the AKT/PKB inhibitor IV. Inhibitors of PKG, GSK, and ROCK did not affect hair bundles, indicating pathways involving these kinases are unlikely to be of importance for the maintenance of hair-bundle structure at this stage of development. The PI3 kinase inhibitors LY294002 and wortmannin had effects that were restricted to the hair bundles, but these were predominantly observed in the more apical regions of cultures where the hair cells are less mature. PI3 kinase-dependent pathways may therefore be more important at earlier stages of hair-bundle development. While the PKA inhibitor H89 caused some disruption of hair-bundle structure this was also only observed in apical-coil cultures and may have been a secondary effect occurring consequent to the vast increase in the surface area of the hair cell’s apical membrane that takes place in response to this compound. By contrast, staurosporine affected hair bundles throughout the entire length of the cochlea, suggesting it was targeting a process fundamental to the maintenance of hair-bundle structure. As cultures prepared from later postnatal mice (P5) also collapse in response to staurosporine treatment, it seems unlikely that the drug is influencing a process that is required uniquely for hair-bundle development.

Staurosporine is a microbial alkaloid that was first isolated in 1977 (Omura et al., 1977). Although it was initially described as a highly potent inhibitor of PKC with IC_50_ values in the low nanomolar range (Tamaoki et al., 1986), it has been since shown to be a broad spectrum, ATP-competitive inhibitor of protein kinases with IC_50_ values ranging from nanomolar to micromolar depending on the protein kinase inhibited (Meggio et al., 1995). The broad range of staurosporine activity is most likely due to its ability to cause induced-fit structural changes in the ATP-binding sites of the kinases (Prade et al., 1997). While staurosporine is known to inhibit a number of kinases including CaM kinase, myosin light chain kinase, PKA, and PKG, it has a very high affinity for PKC and causes hair bundles to collapse at a concentration of just 1 nM, a concentration which is close to its reported IC_50_ (0.7 nM) for PKC. Furthermore, although staurosporine is known to inhibit PKA and PKG, specific inhibitors of these two kinases (H89 and KT5823, respectively) did not cause hair-bundle collapse.

There are a number of PKC isoforms. The conventional Ca^2+^/DAG dependent isoforms (α, β1, β2, γ), the novel DAG dependent/Ca^2+^ independent isoforms (δ, ε, η, θ, and μ) and the atypical isoforms that are neither Ca^2+^ or DAG dependent (ζ, λ). Bisindolylmaleimide I, an inhibitor of the α, β1, β2, γ, δ, and ε isoenzymes had no effect on hair bundles, even at a concentration of 1 μM, nor did a myristoylated pseudosubstrate peptide inhibitor of PKCθ at concentrations above its reported IC_50_. Rottlerin, an inhibitor of the δ and θ isoenzymes (IC_50_ 3–6 μM) had an effect on apical-coil hair bundles at 5 μM but it was toxic at a 10-fold higher concentration. It was therefore hard to determine if the effects observed were similar to those caused by staurosporine. Furthermore, while rottlerin was originally considered to be a PKC δ inhibitor, more recent studies have strongly disputed this and suggest instead that it acts primarily as a mitochondrial uncoupler, disassociating respiration from oxidative phosphorylation, leading to reduction in ATP levels and secondary changes in the function of a multitude of kinase and nonkinase proteins (Soltoff, 2007). Although the effects of rottlerin on hair bundles were of interest, it is not possible to draw clear conclusions about the pathway(s) affected. If the observed effects of staurosporine are indeed due to an inhibition of one of the PKC isoforms, inhibition of the novel isoforms η or μ, or the atypical isoforms ζ and λ, are possibilities remaining to be explored.

The effects of staurosporine may be best explained by the observed loss of the phosphorylated forms of the ERM proteins. These proteins link the actin cytoskeleton to the plasma membrane and are activated by PIP2 and phosphorylation of a critical threonine residue in the C-terminus, a process that unmasks the actin binding domain. Staurosporine treatment has been shown to cause a loss of microvilli from L-cells in vitro, and a concomitant translocation of ERM proteins from the insoluble to the soluble fraction (Yonemura et al., 2002). Radixin was first shown to be a hair-bundle protein in frogs, fishes, and birds by Pataky et al. (2004) and mutations in the *RDX* gene were identified as a cause of nonsyndromic hearing loss at the DFNB24 locus (Khan et al., 2007). A recent analysis (Shin et al., 2013) of the chicken hair-bundle proteome has identified the ERM proteins as the second most abundant class of actin binding proteins present in the hair bundle, and a gene knockout study has shown that a phenotype develops in *Rdx^−^^/^^−^* mice after the onset of hearing at postnatal day 15, a phenotype that includes a fusion of the stereocilia and a detachment of the apical membrane from the cuticular plate (Kitajiri et al., 2004). The lack of a phenotype observed at earlier stages in the *Rdx^−^^/^^−^* mouse is likely to be due to redundancy, as all three ERMs are expressed by hair cells at early stages, with radixin becoming the major ERM protein to be expressed at later stages (Kitajiri et al., 2004). As staurosporine treatment leads to the loss of phosphorylated forms of all three ERM proteins, it is expected that this would lead to a detachment of the hair-cell membrane from the cytoskeleton in cultures of the early postnatal cochlea. Whether different ERMs proteins preferentially mediate membrane-cytoskeletal attachments in discrete domains at the hair cell’s apex remains unclear. Hair-bundle collapse may be driven by both detachment of the apical membrane from the cuticular plate, and a detachment of the plasma membrane from the actin-based core of the stereocilium. Although the latter is not obviously observed, the membrane may progressively unzip from the actin core in the distal direction, i.e., towards the tip of the stereocilium. This would explain the fusion of stereocilia with the apical membrane that is observed by SEM and the thick, presumably fused, stereocilia seen by TEM.

Although the results of this study strongly indicate that the effects of staurosporine are a consequence of the decreased phosphorylation of ERM proteins, reduced phosphorylation of other, as yet unidentified, proteins could also contribute to the observed effect. Furthermore, the kinases that phosphorylate the ERM proteins in the hair bundle and are assumed to be inhibited by staurosporine remain to be identified. While PKCθ (Pietromonaco et al., 1998), PKCα (Ng et al., 2001), and Rho-kinase (Matsui et al., 1998; Oshiro et al., 1998) have all been implicated as ERM kinases in other systems, there are controversies (Matsui et al., 1999) and kinases such as LOK and NIK are known to be involved in ERM phosphorylation in lymphocytes and rat mammary epithelial cells, respectively (Baumgartner et al., 2006; Belkina et al., 2009). The possibility of an alternative hair-bundle ERM kinase cannot, as yet, be excluded. While these issues remain to be resolved, the results of this study clearly indicate that phosphorylation plays a key role in maintaining the structural integrity of the hair bundle of early postnatal sensory hair cells.

## CONFLICT OF INTEREST

The authors declare that they do not have any known conflict of interest.

## AUTHOR CONTRIBUTIONS

All authors contributed to experimentation and discussion of the data. R.J.G. and G.P.R. wrote and constructed the article.

## Supporting Information

Supplementary Information Figure 1. **Activated caspase-3 labelling and TUNEL in staurosporine-treated cultures**. Phalloidin (magenta) and anti-activated caspase-3 (green) staining following treatment with control medium (A) or medium containing 10 nM staurosporine (C) or 0.5 mM neomycin (E) for 18 hours. Images A-C are confocal Z-compressions encompassing a depth of 15 microns below the apical surface and are taken from the mid-apical region. Caspase-3 positive hair cells (arrowheads in E) are only observed in the neomycin condition. Corresponding DAPI-stained nuclei are shown in B, D and F. Although hair-cell nuclei of staurosporine-treated cultures have a normal appearance, those exposed to neomycin are often shrunken and condensed (arrows in F).Phalloidin staining (magenta) and TUNEL (green) following treatment with control medium (G, H) or medium containing 10 nM staurosporine (I, J) or 0.5 mM neomycin (K, L) for 48 hours. Images are confocal Z-compressions encompassing a depth of 15 microns below the apical surface and are taken from the mid-apical region. TUNEL-positive hair-cell nuclei (arrows in L) are abundant following neomycin but not staurosporine. Scale bar = 20 μm.

Supplementary Information Figure 2. **Phospho-ERM levels in staurosporine-treated cochlear cultures**. Immunolabelling with anti-phospho-ERM (A-D) and anti-radixin (E-H) antibodies following incubation in control medium (A-B, E-F) or medium containing 10 nM staurosporine (C-D, G-H) for 5 hours. Phalloidin staining is also shown in panels B, D, F and H. Staurosporine causes a dramatic loss of phospho-ERM (C) but not of radixin labelling (G). (I) Western blots of lysates from cochlear cultures stained for phospho-ERM or radixin following 14 hours in control medium or medium containing 10 nM staurosporine. (J) Quantitative analysis of levels of radixin (magenta bars) and phospho-radixin (green bars) in cochlear cultures that were treated with 10 nM staurosporine for 1, 2.5, 5, 10 and 14 hours. Numbers of independent experiments are indicated. Scale bar in H = 10 μm and applies to A-H.
