# Supplementary figures and images for: Staurosporine-induced collapse of cochlear hair bundles
Source: J Comp Neurol. 2014 Apr 17;522(14):3281–94. doi: 10.1002/cne.23597 (PMC4321040; doi:10.1002/cne.23597)

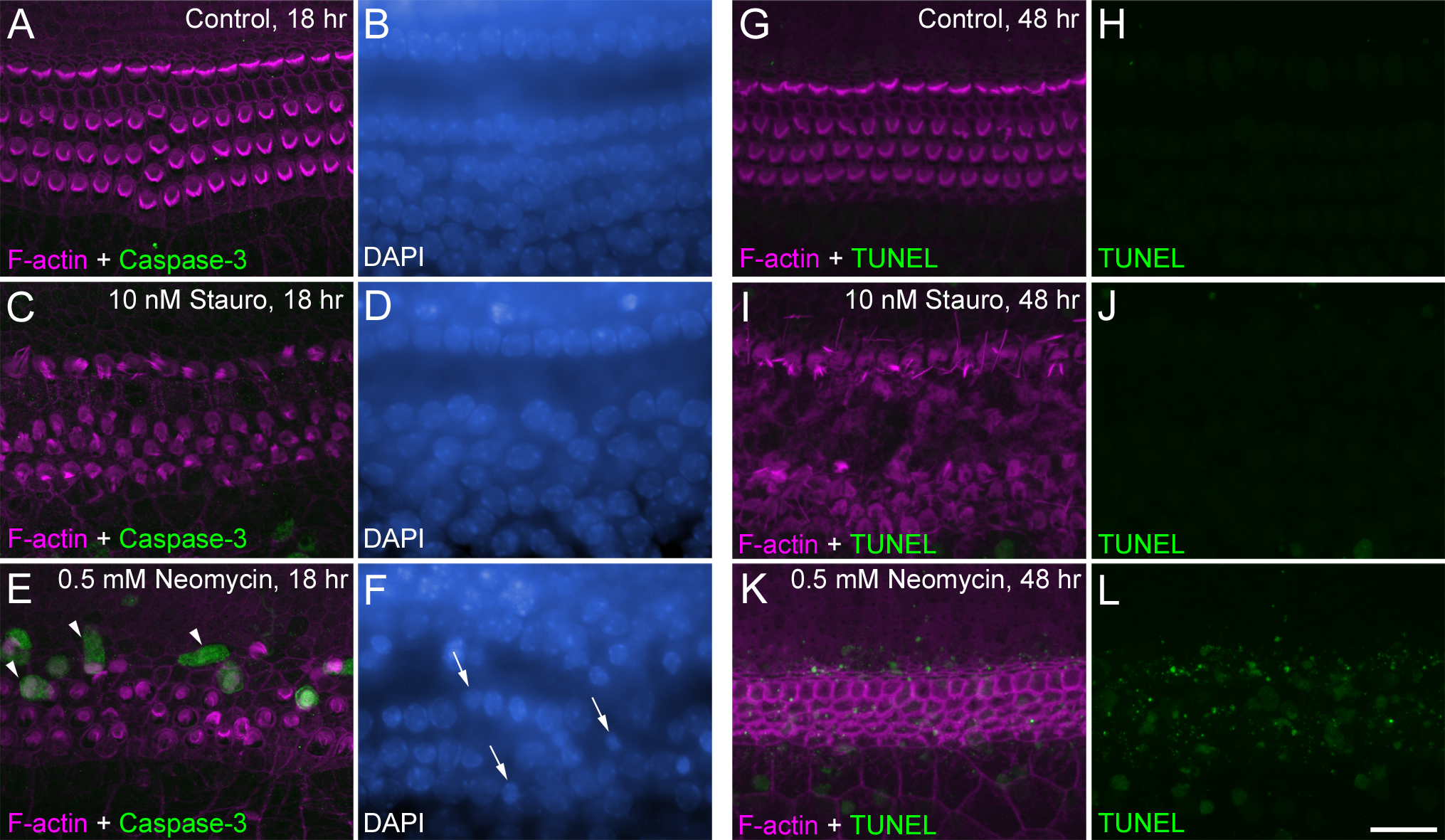

Supplement: Supplementary file 1 — Supplementary Information Figure 1. Activated caspase-3 labelling and TUNEL in staurosporine-treated cultures. Phalloidin (magenta) and anti-activated caspase-3 (green) staining following treatment with control medium (A) or medium containing 10 nM staurosporine (C) or 0.5 mM neomycin (E) for 18 hours. Images A-C are confocal Z-compressions encompassing a depth of 15 microns below the apical surface and are taken from the mid-apical region. Caspase-3 positive hair cells (arrowheads in E) are only observed in the neomycin condition. Corresponding DAPI-stained nuclei are shown in B, D and F. Although hair-cell nuclei of staurosporine-treated cultures have a normal appearance, those exposed to neomycin are often shrunken and condensed (arrows in F).Phalloidin staining (magenta) and TUNEL (green) following treatment with control medium (G, H) or medium containing 10 nM staurosporine (I, J) or 0.5 mM neomycin (K, L) for 48 hours. Images are confocal Z-compressions encompassing a depth of 15 microns below the apical surface and are taken from the mid-apical region. TUNEL-positive hair-cell nuclei (arrows in L) are abundant following neomycin but not staurosporine. Scale bar = 20 μm. [file cne0522-3281-SD1.tif]

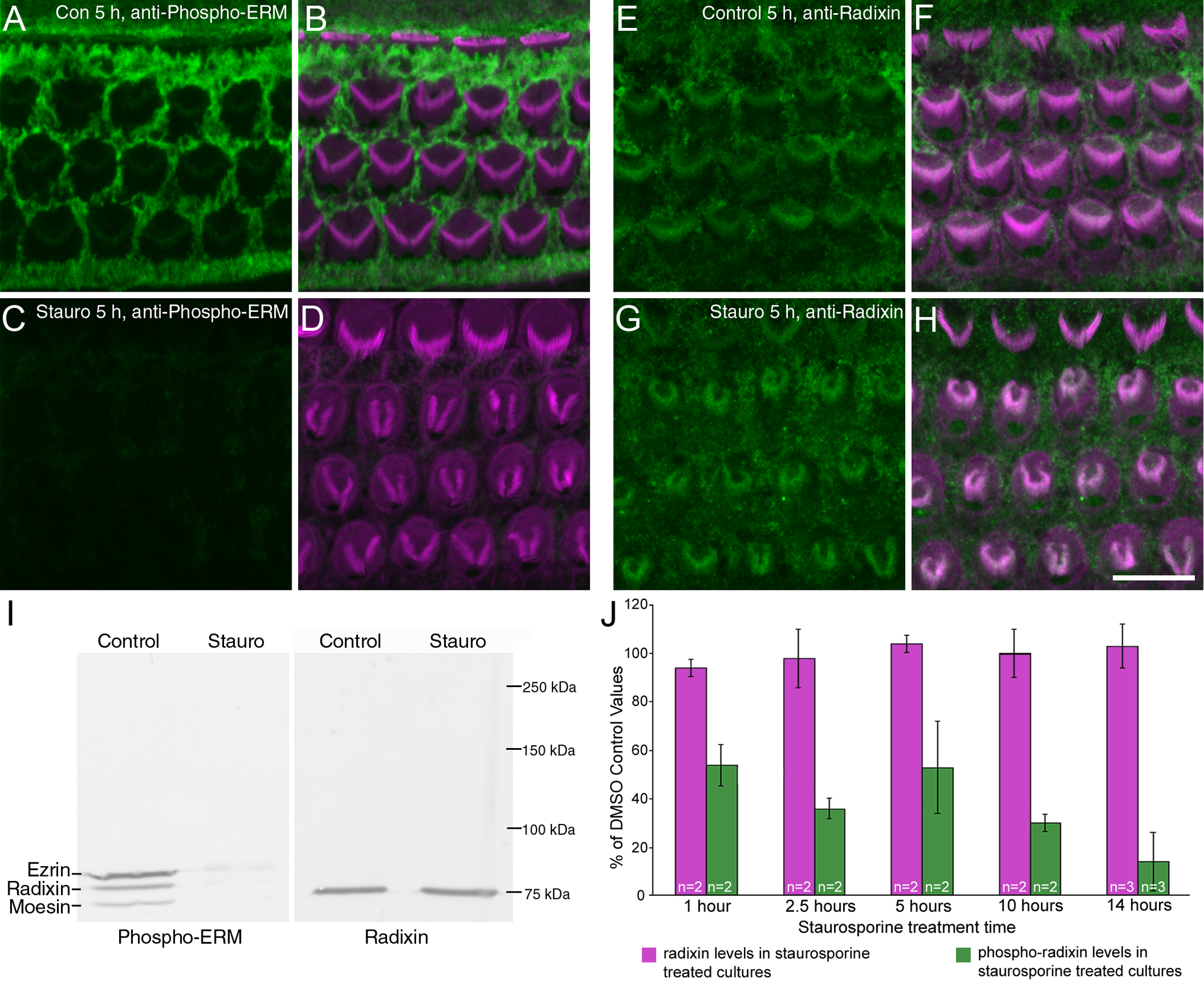

Supplement: Supplementary file 2 — Supplementary Information Figure 2. Phospho-ERM levels in staurosporine-treated cochlear cultures. Immunolabelling with anti-phospho-ERM (A-D) and anti-radixin (E-H) antibodies following incubation in control medium (A-B, E-F) or medium containing 10 nM staurosporine (C-D, G-H) for 5 hours. Phalloidin staining is also shown in panels B, D, F and H. Staurosporine causes a dramatic loss of phospho-ERM (C) but not of radixin labelling (G). (I) Western blots of lysates from cochlear cultures stained for phospho-ERM or radixin following 14 hours in control medium or medium containing 10 nM staurosporine. (J) Quantitative analysis of levels of radixin (magenta bars) and phospho-radixin (green bars) in cochlear cultures that were treated with 10 nM staurosporine for 1, 2.5, 5, 10 and 14 hours. Numbers of independent experiments are indicated. Scale bar in H = 10 μm and applies to A-H. [file cne0522-3281-SD2.tif]
